# Toward the Next Generation of Density Functionals: Escaping the Zero-Sum Game by Using the Exact-Exchange Energy Density

**DOI:** 10.1021/acs.accounts.4c00209

**Published:** 2024-06-21

**Authors:** Martin Kaupp, Artur Wodyński, Alexei V. Arbuznikov, Susanne Fürst, Caspar J. Schattenberg

**Affiliations:** Institut für Chemie, Theoretische Chemie/Quantenchemie, Technische Universität Berlin, Sekr. C7, Strasse des 17. Juni 115, 10623 Berlin, Germany

## Abstract

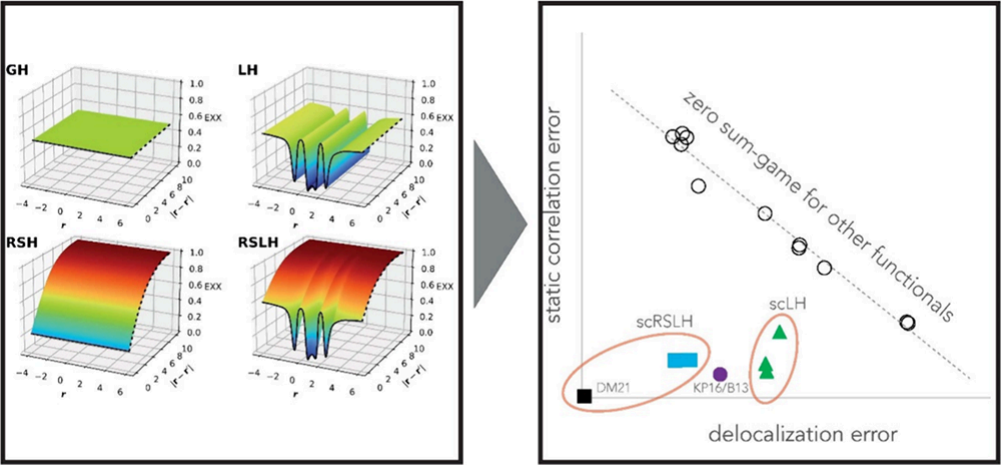

Kohn–Sham density functional theory (KS DFT) is arguably the most widely applied electronic-structure method with tens of thousands of publications each year in a wide variety of fields. Its importance and usefulness can thus hardly be overstated. The central quantity that determines the accuracy of KS DFT calculations is the exchange-correlation functional. Its exact form is unknown, or better “unknowable”, and therefore the derivation of ever more accurate yet efficiently applicable approximate functionals is the “holy grail” in the field. In this context, the simultaneous minimization of so-called delocalization errors and static correlation errors is the greatest challenge that needs to be overcome as we move toward more accurate yet computationally efficient methods. In many cases, an improvement on one of these two aspects (also often termed fractional-charge and fractional-spin errors, respectively) generates a deterioration in the other one. Here we report on recent notable progress in escaping this so-called “zero-sum-game” by constructing new functionals based on the exact-exchange energy density. In particular, local hybrid and range-separated local hybrid functionals are discussed that incorporate additional terms that deal with static correlation as well as with delocalization errors. Taking hints from other coordinate-space models of nondynamical and strong electron correlations (the B13 and KP16/B13 models), position-dependent functions that cover these aspects in real space have been devised and incorporated into the local-mixing functions determining the position-dependence of exact-exchange admixture of local hybrids as well as into the treatment of range separation in range-separated local hybrids. While initial functionals followed closely the B13 and KP16/B13 frameworks, meanwhile simpler real-space functions based on ratios of semilocal and exact-exchange energy densities have been found, providing a basis for relatively simple and numerically convenient functionals. Notably, the correction terms can either increase or decrease exact-exchange admixture locally in real space (and in interelectronic-distance space), leading even to regions with negative admixture in cases of particularly strong static correlations. Efficient implementations into a fast computer code (Turbomole) using seminumerical integration techniques make such local hybrid and range-separated local hybrid functionals promising new tools for complicated composite systems in many research areas, where simultaneously small delocalization errors and static correlation errors are crucial. First real-world application examples of the new functionals are provided, including stretched bonds, symmetry-breaking and hyperfine coupling in open-shell transition-metal complexes, as well as a reduction of static correlation errors in the computation of nuclear shieldings and magnetizabilities. The newest versions of range-separated local hybrids (e.g., ωLH23tdE) retain the excellent frontier-orbital energies and correct asymptotic exchange-correlation potential of the underlying ωLH22t functional while improving substantially on strong-correlation cases. The form of these functionals can be further linked to the performance of the recent impactful deep-neural-network “black-box” functional DM21, which itself may be viewed as a range-separated local hybrid.

## Key References

- WodyńskiA.; ArbuznikovA. V.; KauppM.Strong Correlation Density Functionals Made Simple. J. Chem. Phys.2023, 158, 244117.37387453 10.1063/5.0153463(1) An earlier, more complicated framework for applying strong-correlation functions to the exact-exchange admixture in local hybrid functionals has been simplified based on simple ratios of exchange-energy densities, providing the basis for transferring such ideas to range-separated local hybrids (see below).
- FürstS.; KauppM.; WodyńskiA.Range-separated local hybrid functionals with small fractional-charge and fractional-spin errors: escaping the zero-sum game of DFT functionals. J. Chem. Theory Comput.2023, 19, 8639–8653.37972297 10.1021/acs.jctc.3c00877(2) Based on the accurate ωLH22t range-separated local hybrid, functionals with correction terms for strong correlations and for delocalization-errors are constructed rationally, providing the so far most significant escape of routinely applicable density functionals from the usual zero-sum-game.
- WodyńskiA.; LauwB.; ReimannM.; KauppM.Revisiting spin symmetry breaking and hyperfine couplings in transition-metal complexes using density functionals based on the exact-exchange energy density. J. Chem. Theory Comput.2024, 20, 2033–204838411554 10.1021/acs.jctc.3c01422PMC10938646.^3^ The first real-life applications of the previously published range-separated local hybrids to a series of open-shell manganese complexes show that both strong-correlation and delocalization-error terms can help minimize spin-symmetry breaking and thereby improve hyperfine couplings.
- SchattenbergC. J.; KauppM.Implementation and first evaluation of strong-correlation-corrected local hybrid functionals for the calculation of NMR shieldings and shifts. J. Phys. Chem. A2024, 128, 2253–227138456430 10.1021/acs.jpca.3c08507PMC10961831.^4^ Strong-correlation-corrected local hybrids have been implemented into an efficient code for the calculation of nuclear shieldings (Turbomole) and were evaluated on two large and representative test sets of main-group and transition-metal NMR shieldings/shifts, respectively.

## 1 Introduction

As we strive for an ever more detailed atomistic picture of molecular and condensed-phase sciences, the need for accurate electronic-structure methods applicable to relatively large systems continues to be a central topic of current research. While developments toward low-scaling correlated ab initio quantum-chemical methodologies based on local correlation approaches are moving forward apace (see, e.g. refs (5,6) and references therein), Kohn–Sham density functional theory (KS DFT) retains its advantage regarding the applicability to larger systems and is thus, and likely will continue to be for some time, the most widely used quantum-chemical power house.^7^ Less demanding but also less accurate semiempirical MO methods, often parametrized against DFT, then fill a gap toward still larger system sizes.^8^

The central quantity that determines the achievable accuracy of KS DFT is the exchange-correlation (XC) functional (and its functional derivatives). Improving the accuracy of approximate XC functionals (density functional approximations, DFAs) toward the exact solution of the Schrödinger equation or its relativistic extensions is not as straightforward as it is to systematically improve the accuracy of post-HF approaches. Our focus here will be on the main challenges that the development of DFAs has faced and still in part faces: a) earlier “standard functionals” did not cover dispersion interactions; b) often self-interaction errors (SIE) plague approximate functionals; c) we need to be able to treat systems with strong static correlation. Notwithstanding the need for further improvements, for all practical purposes point a) seems under control, either by adding atom-additive dispersion corrections^9^ or by explicit nonlocal van-der-Waals functionals.^10^ Points b) and c) are thus the major remaining challenges. They have been discussed in terms of a “zero-sum-game” of DFA development.^11−14^ That is, functionals that aim to reduce SIEs by admixing more exact exchange (EXX) in so-called hybrid functionals (see below) tend to suffer from larger static correlation errors. Functionals with low or zero EXX admixtures tend to perform somewhat better for strong-correlation cases but suffer from larger SIEs. It is easy to envision molecular or condensed-phase systems for which low SIEs are crucial in one part of the system but strong correlations may be important in another part. This account will focus on new approaches to escape this foundational zero-sum-game of DFAs by using the local EXX energy density in various ways. Notably, our focus is on the fourth, “hyper-GGA”, rung of Perdew’s ladder (“Jacob’s ladder”) hierarchy.^15^ This rung is arguably the most important one in many applications in chemistry, and increasingly also in solid-state physics and material science. The fifth, “fully nonlocal” rung, which includes the double-hybrid functionals (DHs)^16,17^ or the so-called σ-functionals,^18^ has the potential to provide even higher accuracy, but it involves computationally more demanding wave function or RPA-type correlation contributions, and for many properties such DFAs are limited to smaller systems. The first to third rung (local density approximation – LDA – generalized gradient approximation – GGA – and meta-GGA, respectively) offer less room for improvement. Notably, the ideas discussed here for the fourth rung may also be extended to the fifth rung. We note in passing that methods exist where a multiconfigurational wave function to cover static correlation is combined with some DFT contribution for dynamical correlation, either using range separation^19−21^ or by other means.^22,23^ Such approaches clearly can escape the zero-sum game but are outside the scope of this article. They are also computationally more demanding and typically not usable in a black-box way.

## 2 Background

### 2.1 Fractional Charge and Fractional Spin Errors

The discussion of the above-mentioned zero-sum game between SIEs and static correlation errors has often be coined alternatively within the framework of fractional charge errors^24^ (FCEs) and fractional spin errors (FSEs),^25^ i.e. by dealing with artificial fractional electron occupations. FCEs are related to SIE and to so-called delocalization errors^26^ of many approximate functionals, while FSEs allow a quantification of static correlation errors. Approximate functionals typically exhibit both appreciable FCEs and FSEs. The above-mentioned zero-sum game means that larger EXX admixtures tend to reduce FCEs but increase FSEs.^11−13,27^ It is important to note that atomic FSEs (energy differences between a spin-polarized and fully -depolarized atom) give one-half of the static correlation errors at the dissociation limit for the spin-restricted bond dissociation curve of the corresponding diatomic system.^28^ Note that the straight-line behavior of the exact functional as a basis for FCEs has recently been put in question by Baerends who showed that arguments based on a thermodynamic model underpinning Janak’s theorem and related aspects of fractional charges are not valid.^29,30^ We will thus in most cases prefer to speak of delocalization errors^26^ rather than FCEs while maintaining the use of the term FSE.

### 2.2 Hybrid Functionals and Range Separation

**Figure 1:**
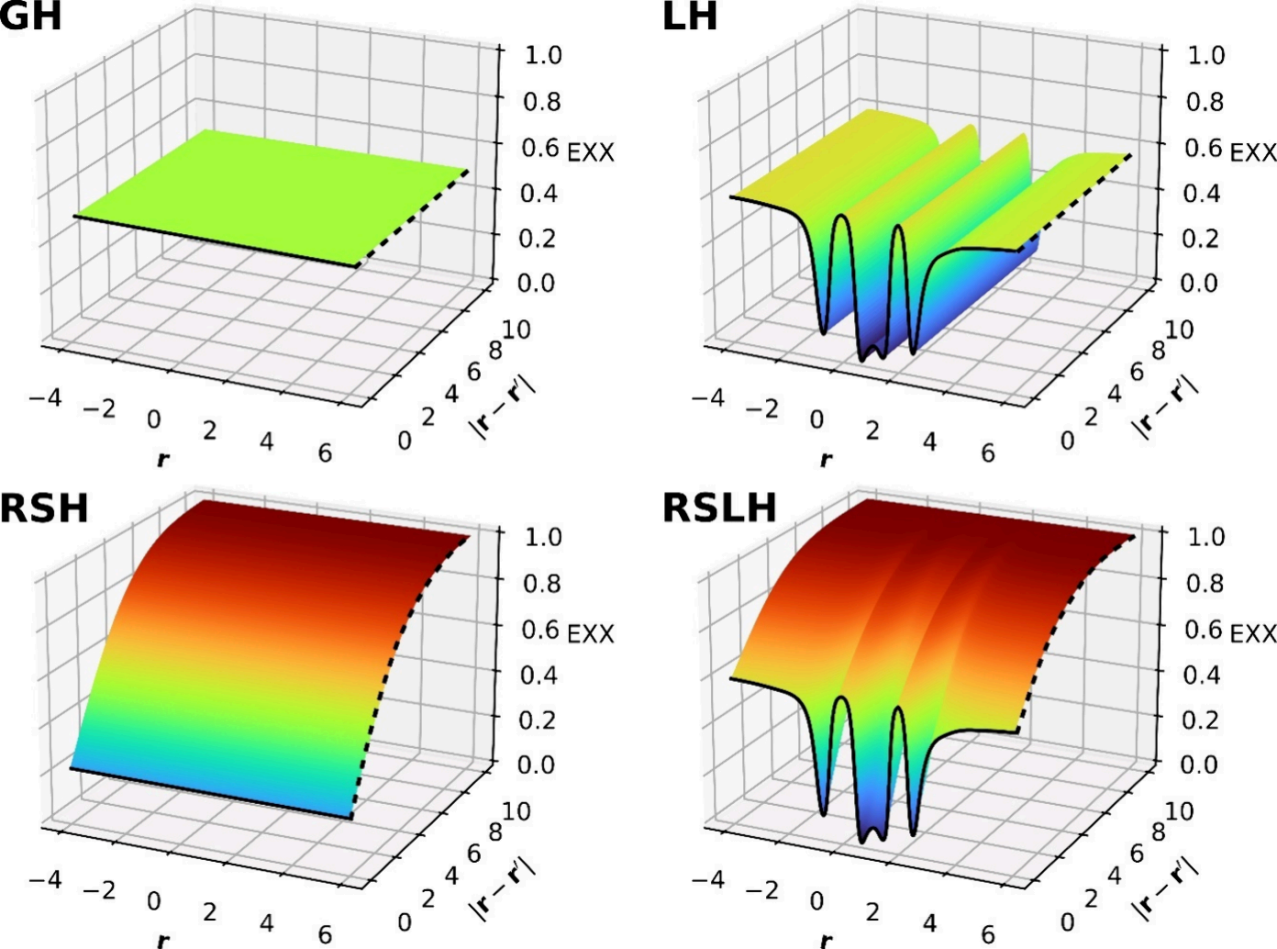
EXX admixture in different types of hybrid functionals in real space (shown for the example of a bond direction in a diatomic molecule) and in interelectronic distance space. The real-space dimension is represented by the bond axis in a CO molecule, and the examples shown correspond to the BHLYP GH, the LH20t LH, the ωB97X-D RSH, and the ωLH22t RSLH. Reprinted with permission from ref (35), copyright 2023, American Chemical Society.

Rung 4 on the DFT ladder was essentially brought to life by Becke’s introduction of a constant admixture of Hartree–Fock exchange (see, e.g., Figure 1, top left), i.e. by the introduction of global hybrid functionals (GHs),^31^ as exemplified by the popular B3LYP-type DFAs.^32^ The limitations of GHs have been widely discussed, for example regarding the need to use different EXX admixtures (a_0_ values) for different systems or properties.^7^ Nevertheless, it is probably fair to say that GHs are still the overall most widely used XC functionals in chemistry, and the DHs on rung 5 (see above) are also most often extensions of such GHs. One extension of hybrid functionals important in its own right, as well as for the recent developments discussed below are “range-separated hybrids” (RSHs) that vary the EXX admixture along the interelectronic distance for each electron pair in the system.^33^ Here we emphasize long-range corrected (LC) functionals that mix in LR HF exchange to improve in particular the TDDFT treatment of electronic excitations with charge-transfer (CT) character and related quantities, but they are also effective in minimizing delocalization errors.^34^ LC functionals are probably the most important and most widely used RSH examples (see also below), and the dependence of their EXX admixture on interelectronic distance can be inferred from Figure 1 (bottom left).

### 2.3 Local Hybrid Functionals

Local hybrid functionals (LHs) are at the core of the developments covered here. The state of the art in 2019 on LHs has been comprehensively reviewed.^36^ We refer the reader to that review for further details and mention only some salient aspects. While the above-mentioned GHs, RSHs or DHs generally mix spatially integrated exact exchange with some semilocal exchange energies, this account focuses on functionals based on the local exact-exchange energy density, mixed with other ingredients in a position-dependent, real-space manner. LHs vary EXX admixture in real space, governed by a local mixing function (LMF, Figure 1, top right).^36,37^ While conceptual problems arising from the ambiguities of exchange-energy densities, the so-called “gauge problem” (see below), had been discussed in the late 1990s,^38^ the first explicit LH was put forward in 2003 by Jaramillo et al.^37^ The initial functionals ignored the above-mentioned ambiguity. In that case, the best performance was achieved with LHs based on a mixing of LDA and EXX energy densities.^36^ One early exception was the PSTS functional,^39^ which a) was based on TPSS meta-GGA exchange, and b) for which relatively complicated “calibration functions” (CFs) were proposed to deal with the gauge problem.^40^ A general LH may be written as^36^
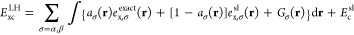
1where *a*_σ_(**r**) is the LMF (for unrestricted open-shell calculations we distinguish spin-channel LMFs, *a*_α_(**r**) ≠ *a*_β_(**r**), or the more successful common LMFs, *a*_α_(**r**) = *a*_β_(**r**) ≡ *a*(**r**)). *e*_x,σ_^exact^(**r**) is the EXX energy density. It is defined in terms of the molecular orbitals (MOs) φ_p,σ_^*^(***r***) and the ground-state spin-density matrix *D*^σ^ as
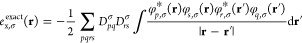
2with *p*, *q*, *r*, *s* being general MO indices, and *D*_*pq*_^σ^, *D*_*rs*_^σ^ the corresponding σ-spin density-matrix elements. *e*_x,σ_^sl^(***r***) is the semilocal exchange-energy density and *G*_σ_*(***r***)* is the CF. *E*_*c*_^sl^ is a (typically semilocal) correlation functional. A reformulation of eq 1 that is particularly suitable for this account is^15,36^
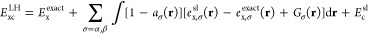
3That is, we start from full exact exchange together with semilocal correlation and interpret the middle term as a nonlocal correlation term, sometimes associated with nondynamical correlation (NDC). This links LHs to other coordinate-space models of NDC, such as Becke’s B05 *ansatz*(41) (see below).

While early implementations of LHs were inefficient, newer efficient implementations^42−48^ based on seminumerical integration techniques render the computational demands of LH calculations comparable to those of GHs or RSHs. The implementation within the efficient framework of the Turbomole code^49^ currently encompasses by far the most extensive functionality for LHs, including ground-state energies^43^ and gradients,^44^ TDDFT excited-state energies,^45^ gradients^50^ and properties,^51^ and a wide range of NMR and EPR parameters and magnetic properties,^4,48,52−56^ including quasirelativistic two-component treatments that include spin–orbit coupling.

The local admixture of EXX and semilocal exchange-energy densities introduces the above-mentioned gauge problem of LHs, as two energy densities are mixed locally. While addition of a calibration function (CF) that integrates to zero over space does not affect the XC energy for GHs or RSHs, it does so for LHs and related functionals, due to the multiplication of the integrands in eqs 1 and 3 by the LMF or its complement.^36,40^ As the sophisticated CF suggested for the PSTS functional^40^ (see above) poses substantial computational difficulties, we introduced semilocal CFs that can be derived from any semilocal exchange functional by successive partial integration steps (“partial integration gauge”, pig), so far implemented up to second order (pig2) for GGA exchange.^57^

These developments led to the LH20t functional,^58^ which provides the starting point for the newer work described in this account. LH20t uses a so-called t-LMF, i.e. a scaled ratio between the von Weizsäcker and Kohn–Sham kinetic-energy densities, to admix locally EXX to PBE exchange, augmented by a pig2 CF^57^ and complemented by a reoptimized B95c meta-GGA correlation functional.^59^ When combined with D3 or D4 dispersion corrections, LH20t has been demonstrated to outperform essentially all regular GHs for the large GMTKN55 test suite of general main-group thermochemistry, kinetics and noncovalent interactions,^60^ indicating that the gauge problem has been largely eliminated by the pig2 CF.^58^ A wide variety of further tests on transition-metal organometallic^61^ and mixed-valence oxo test sets, as well as on electric^51^ and magnetic^53,56,62,63^ properties and a wide variety of excitation-energy calculations at TDDFT level^64−67^ confirm LH20t to be a competitive general-use functional. Notably, however, it does not account accurately for static correlation, i.e., it exhibits sizable FSEs (see below). Additionally, the t-LMF does not exhibit the correct coordinate scaling in the high-density limit, and its performance for core-related properties like core-excitation or -ionization energies therefore deteriorates as we move to heavier nuclei. The recent LH23pt functional has introduced an LMF with an improved core part. It retains the high quality of LH20t for 1s core excitation and ionization energies of second-period elements but improves substantially for third-period (and fourth-period) atoms.^68^ We note in passing attempts to construct functionals that escape the zero-sum game by modeling the position-dependent EXX admixture using so-called rung 3.5 ingredients.^69,70^

### 2.4 Coordinate-Space Models of Nondynamic and Strong Correlations

A separate and independent DFA development based on the EXX energy density with bearing on the newer developments described below is Becke’s B05 coordinate-space model of NDC.^41^ It can be written as
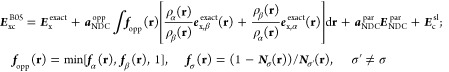
4The middle nonlocal correlation term (second line, the less important parallel-spin contribution is not detailed here^41^) deepens the EXX hole in areas of space, where the effective normalization of the projection of the EXX hole onto a model hole indicates appreciable NDC. A reverse version of the model BR89x exchange hole^71^ (revBR machinery) is used here. Due to their structure, B05 and related functionals are difficult, albeit not impossible,^72^ to use routinely in self-consistent calculations. The similarity of eqs 3 and 4 provides an important link between LHs and the B05 model, which we exploit (see below).

The B05 model can be combined with dispersion terms (after correcting for unphysical repulsion contributions similar to the gauge problem of LHs, see above), and it has been used as an ingredient of further functionals with increasing parametrization and increasingly accurate performance for, e.g., GMTKN55 energetics.^73,74^ These functionals share, however, the problems of B05 regarding self-consistent computations. More importantly, Becke noted that B05 does not cover strong correlations, as it exhibits appreciable FSEs. He argued that the NDC term of B05 includes only the potential energy of NDC and misses the kinetic-energy correlation contribution.^75^ The latter has to be recovered from a local version of the adiabatic connection (AC), i.e. from an integration of the energy densities over the coupling strength parameter in a many-electron system. This gave rise to the B13 functional, where an additional B13_strC_ term has been included.^75^ B13 is arguably the first rung 4 functional that escapes to a significant extent the zero-sum game. Subsequently, Kong and Proynov suggested a different way in which the local AC can be formulated:^76^ a multiplicative strong-correlation function *q*_*AC*_(**r**) is applied to the integrand of the B05-type NDC term, eq 4. This leads to the KP16/B13 functional, which also diminishes FSEs compared to B05.^76^ We found this model, in particular the multiplicative function *q*_*AC*_(**r**), particularly suitable to transfer the ideas of coordinate-space models of strong correlation to the LH framework when comparing the middle terms of eqs 3 and 5).
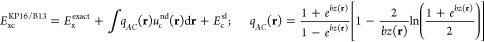
5Notably, *q*_*AC*_(**r**) depends on coordinate-space function *z*(**r**) that determines the importance of strong correlations in real space. In KP16/B13, *z*(**r**) is the ratio of NDC and DC (dynamical correlation) potentials built from the revBR machinery underlying B05.^76^

## 3 Introduction of Strong-Correlation Terms into Local Hybrids

**Figure 2:**
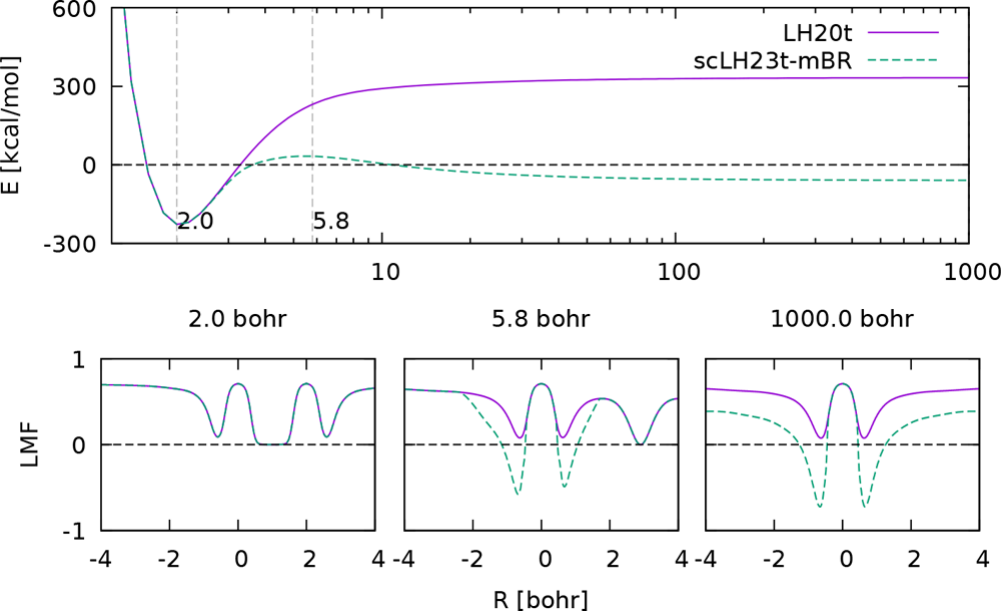
Top: Spin-restricted bond dissociation curves for the N_2_ molecule with LH20t and the related strong-correlation-corrected scLH23t-mBR. Bottom: Comparison of the t- and tq-LMFs of LH20t and scLH23t-mBR, respectively, at equilibrium distance (2.0 bohr), at intermediate distance (5.8 bohr), and at dissociation (1000.0 bohr). At equilibrium distance, both N atoms are visible; only the region around one N atom is seen in the LMF-plots at larger distances.

These coordinate-space models exhibit promising concepts, while the functionals themselves suffer from the numerical difficulties of the B05 NDC term. It appeared logical to apply a KP16/B13-style strong-correlation function *q*_*AC*_(**r**) within the LH framework. Exploratory work for a simple LDA-based LH without a CF^77^ indicated a) better performance by adiabatically connecting both the middle NDC term and the DC term with *q*_*AC*_(**r**), and b) direct use of the NDC expressions of such a simple LH to construct *q*_*AC*_(**r**) does not provide the desired substantial reduction of FSEs. This led initially to a “hybrid approach” where *q*_*AC*_(**r**) is derived within a modified KP16/B13 framework and then applied to the LH correlation terms. The initial results for the scLH21ct-SVWN-m functional^77^ were promising, so that the work was extended^72^ to correcting the more advanced LH20t functional (see above). The final structure of such strong-correlation-corrected LHs (scLHs) is
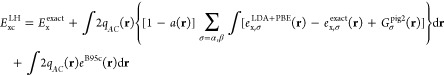
6It turned out that by damping *q*_*AC*_(**r**) for small values of *z*(**r**), double-counting of NDC contributions within the LH framework could be minimized, and the excellent performance of LH20t for weakly correlated situations was preserved (e.g., regarding GMTKN55 energetics, see also section 6). This led to the scLH22t functional.^72^ It provides essentially LH20t accuracy for weakly correlated situations, while reducing FSEs to a similar extent as KP16/B13 (an “undamped” version, scLH22ta, was also reported^72^). The way in which *q*_*AC*_(**r**) brings in strong correlations is demonstrated in Figure 2 (using the more recent scLH23t-mBR^1^) for the spin-restricted bond dissociation curve of the N_2_ molecule, where in the lower panels the overall LMF including *q*_*AC*_(**r**) (“tq-LMF”) is compared to the unmodified t-LMF of LH20t for different internuclear distances. While the LMF is essentially unchanged at equilibrium distance, a local reduction is apparent for a stretched bond (at 5.8 bohr). At the dissociation limit (1000.0 bohr), strong correlations are mimicked by having even larger locally negative EXX admixture in the tq-LMF, providing a substantially improved energy.^1,72^

In subsequent work,^1^ detailed analyses led to appreciably simplified formulations of *z*(**r**) and *q*_*AC*_(**r**): we can bypass the complicated exchange-hole normalization involved in B05 (eq 4) and, e.g., in KP16/B13, and construct *z*(**r**) simply from the ratio of a semilocal and the EXX energy density. This brings us much closer to the goal of a closed scLH formulation. The evaluations showed^1^ that a modified BR89x^71^ energy density (mBR model) provided the best results, but even a simple formulation based on the PBE GGA is possible. Different mathematical interpolations for the local AC were tested. Combining an error-function mapping and the mBR energy density led to the scLH23t-mBR functional,^1^ which performed similarly well as the much more involved scLH22t functional.^72^ A more complex Padé form of *q*_*AC*_(**r**) allowed a reduction of the unphysical local maxima exhibited by essentially all coordinate-space models of strong correlation in many spin-restricted bond dissociation curves (see Figure 2), by fitting the adjustable parameters of *q*_*AC*_ to a multireference CI dissociation curve of the N_2_ molecule.^1^

## 4 Range-Separated Local Hybrids: The ωLH22t Functional

One area where LHs clearly cannot compete with the best LC RSHs are excitations with substantial charge-transfer character in linear-response-TDDFT. Here those RSHs that exhibit 100% LR EXX admixture, and thus have the correct long-range asymptotic exchange-correlation potential, clearly perform best. Real-space EXX admixture in LHs can give the correct LR exchange-energy density but not the correct potential.^78^ RSHs can provide this information via the interelectronic-distance dependence of their EXX admixture (Figure 1).^79^ But as their separation between SR DFT and LR EXX contributions typically is described just by one constant range-separation parameter ω, the lack of flexibility becomes a problem. That is, the optimal ω depends significantly on chromophore size,^80,81^ but also differs even for different elements in the periodic table.^82^ This has led to the emergence of so-called optimally tuned RSHs (OT-RSHs) where typically for each system an optimal ω is determined from its ionization-potential (IP) theorem (that is, matching the IP obtained self-consistently and that from Koopmans’ theorem; see, e.g., refs (82−84)). The disadvantage of this “non-empirical tuning” is, that with different ω values obtained for different systems we also have different functionals in each case. This lack of size consistency may affect, e.g., the smoothness of computed potential-energy surfaces,^82^ or the treatment of molecule–surface reactions, where we may typically expect different optimal ω values for, e.g., a metal surface and an adsorbed molecule.^85^ And tuning for one particular IP may not provide good results for other ionizations.^86^

Apart from using RSHs with some SR EXX admixture,^87^ one remedy can be to replace the constant ω by a range-separation function depending on position in space. This leads to the concept of locally range-separated hybrids (LRSH).^85,88−92^ Initially, outer-valence spectra with comparable accuracy as for OT-RSHs could indeed be obtained.^85^ Using an advanced range-separation function and a self-interaction-corrected correlation functional,^89^ improved ground-state computations compared to regular RSHs could be demonstrated,^90^ and most recently, an LRSH with excellent performance for band gaps has been reported.^92^

Another way to combine the LH and RSH ideas is the position-dependent mixing of SR and LR exchange-energy densities by an LMF, leading to range-separated local hybrids (RSLHs), see Figure 1, bottom right.^93,94^ We need to distinguish this concept from the use of range separation in the correlation functional of LHs, which we have also employed elsewhere.^95^ Based on a recent efficient seminumerical Turbomole implementation,^35^ a modern RSLH, ωLH22t (see eq 7), has been optimized,^35^ which is essentially an RSLH extension of the LH20t functional.
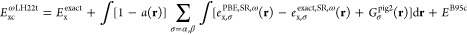
7That is, it mixes SR PBE and SR exact exchange by a t-LMF in the middle term, augmented by a pig2 CF^57^ and by reoptimized B95c correlation. ωLH22t improves further upon LH20t for GMTKN55 main-group energetics evaluations and is among the best-performing rung 4 functionals overall.^35^ It also is competitive for organometallic reaction energies and barriers (see above for the discussion with regard to LH20t) and improves upon typical potential-energy curves sensitive to delocalization errors. The LR EXX admixture (which arises from full EXX in the first term and the subtracted SR-EXX contributions in the middle term) improves performance compared to LH20t for a wide variety of inter- and intramolecular charge-transfer excitations while retaining largely the advantages of LHs with common t-LMF for triplet excitations, and for core excitations.^35^

In particular, recent evaluations^96^ of ωLH22t showed that it provides excellent frontier orbital energies. That is, ionization potentials, electron affinities, and fundamental band gaps can be obtained from Koopmans’ theorem for a wide variety of systems, from atomic anions to large chromophores of importance in molecular electronics and organic photovoltaics, without the need for system-dependent tuning (see also section 6 below). Excellent outer-valence spectra are obtained.^96^ This reflects the flexibility added by the position-dependent SR EXX admixture and renders ωLH22t highly attractive for a wide variety of application areas. However, due to its 100% LR EXX admixture, ωLH22t exhibits substantial FSEs that are even about a factor of 1.5 larger than those of LH20t.^2^

## 5 Range-Separated Local Hybrids with Strong-Correlation Terms

The question thus arises if we can transfer the ideas of scLHs as elaborated above to the case of ωLH22t. One indication that this should be possible is provided by the recently reported DM21 (“deep mind 21”) functional.^97^ It consists of a deep neural network, DNN, trained on various molecular and atomic energies, with particular attention to minimizing FCE and FSE. The authors stated that DM21 (which is augmented by D3 dispersion corrections) essentially is an RSLH, given the features “handed” to the neural network. Yet, the black-box nature of the DNN with tens of thousands of parameters does not allow any insights into the detailed form of the functional. Notably, DM21 achieves low FSEs as well as low FCEs for which it has been specifically trained (see also section 6 below). Apart from its black-box character, the numerical complexity of DM21 poses some challenges for routine applications,^97^ for example regarding SCF convergence with transition-metal systems.^98^

We have recently transferred the idea of sc-corrections to the ωLH22t RSLH.^2^ When writing ωLH22t as in eq 7, the integrand of the middle NDC term features the difference of only the SR parts of semilocal and exact-exchange energy densities. As the kinetic energy of strong correlation that we want to recover is expected to be of LR character, applying *q*_*AC*_(**r**) directly to this form is not expected to be sufficient, as borne out numerically.^2^ Therefore, an additional contribution has been introduced into the integrand of the middle term, governed by a strong-correlation switching function *f*_FR_(**r**) depending also on *z*(**r**), just like *q*_AC_(**r**).
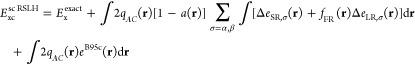
8with
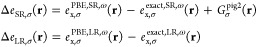
9*f*_FR_ introduces LR contributions in regions of space where *z*(**r**) becomes significant, eq 8, i.e. in the very same regions where *q*_AC_(**r**) becomes appreciably larger than 0.5. While *q*_AC_(**r**) ranges from 0.5 in the absence to 1.0 in the maximal presence of strong correlations, *f*_FR_(**r**) simultaneously goes from 0.0 to 1.0. The forms of *f*_FR_(**r**) and *q*_AC_(**r**) and their adjustable parameters are chosen to be essentially the same, so that the empiricism of the functional is not increased. This indeed paves the way to successful scRSLHs that largely retain the excellent performance for weakly correlated systems and low delocalization errors of ωLH22t, while reducing dramatically FSEs (see section 6 for data).^2^ The additional term depending on *f*_FR_(**r**) in fact reduces LR EXX contributions locally in regions of space where strong correlations are detected.

Several different forms were again constructed, including an error-function form as in scLH23t-mBR, leading to ωLH23tE, and a Padé form.^2^ To improve performance further, an additional correction term to the LMF to reduce delocalization errors (DEs) in abnormal open-shell cases was evaluated. It was motivated by the so-called “*a*_2_ term” in the LMF of the PSTS LH^39^ but depends also on *z*(**r**). However, while *q*_*AC*_(**r**) and *f*_*FR*_(**r**) are meant to govern strong-correlation effects, the “DE correction” term in the new “td-LMF” reduces DEs in certain abnormal open-shell situations.^2^

The resulting functionals, of which we discuss here ωLH23tdE, indeed improve over their uncorrected variants, as well as over ωLH22t. Inclusion of a DE term into ωLH22t without sc-corrections leads to ωLH23td, which also improves over the underlying ωLH22t RSLH for open-shell cases with significant SIE. The scRSLH functionals are significant steps on the way outside the usual zero-sum game. In particular, they come very close to the outstanding performance of ωLH22t for using Koopmans’ theorem to extract quasiparticle energies for a wide range of systems and chromophores (see also section 6).^2^ As a rare downside, it was found that the sc-corrections overshoot appreciably for the hydride anion (H^–^), due to significant SIE within the semilocal exchange-energy densities entering *z*(**r**).^2^

## 6 First Real-Life Tests of the New Functionals

**Figure 3:**
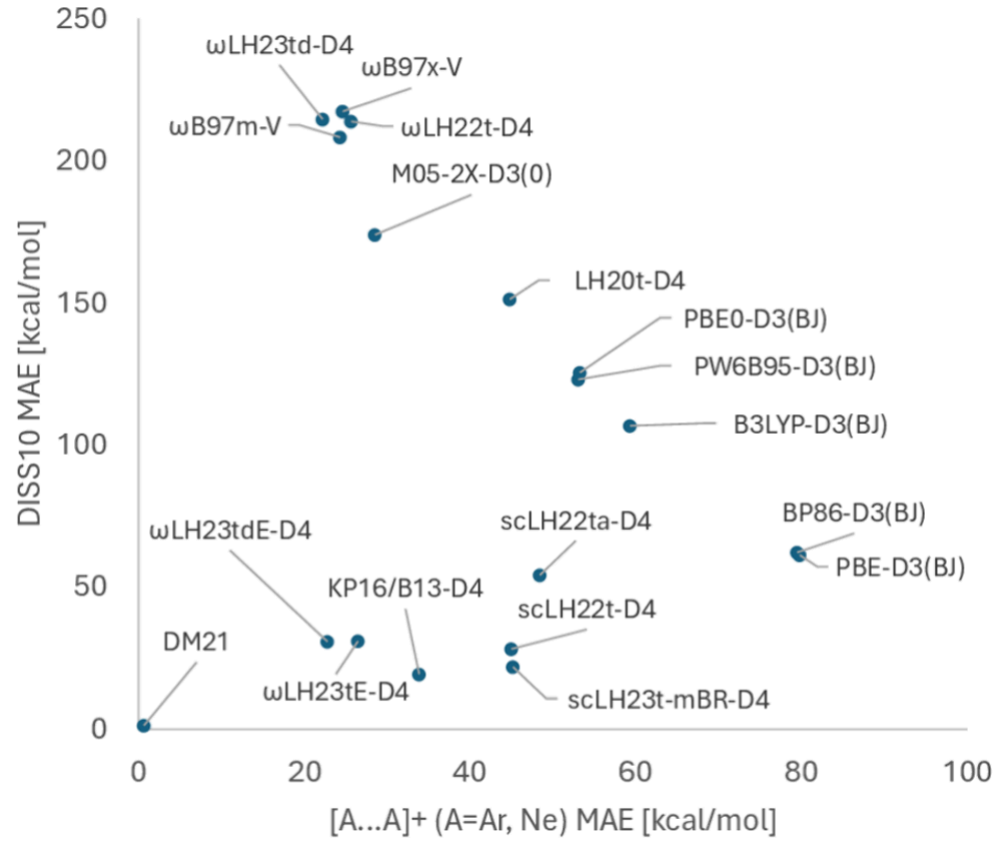
Evaluation of the zero-sum game between FSEs and delocalization errors for different functionals using the MAEs of the DISS10 set as a measure of the former and the MAEs for the dissociation limits of the Ne_2_^+^ and Ar_2_^+^ radical cations as measure of the latter.

We start this section by discussing to what extent the new functionals with sc- and DE-terms have already escaped the zero-sum game between improving upon static correlation errors (FSEs) and delocalization errors. Alternatively, we may ask how well the sc-corrected functionals perform for weakly correlated systems, e.g., for GMTKN55 energetics. Extensive numerical comparisons to answer these questions are available in the original publications^1,2,72,77^ A compact representation of such data suggested by Janesko^11^ is to plot quantities related to FSEs against those related to FCEs. Figure 3 provides such a comparison by plotting the mean absolute errors (MAEs) of several state-of-the-art rung 4 functionals (and of two GGAs on rung 2) for the DISS10 set of the asymptote of the spin-restricted binding curves for ten homonuclear main-group diatomics^72^ as a good measure of FSEs against the MAEs of the asymptotes of the (spin-unrestricted) binding curves of the Ne_2_^+^ and Ar_2_^+^ radical cations as typical measures of delocalization errors. Most functionals, including many not included in the plot, fall roughly on a line with negative slope that illustrates the zero-sum game: an improvement for DISS10 worsens the performance for the radical cations. This includes LH20t, ωLH22t or ωLH23td. The latter two have small delocalization errors but appreciable FSEs. Only functionals with sc-corrections escape this dilemma. The DM21 deep neural network exemplifies the essentially perfect behavior in this case. This should not be too surprising, as DM21 has been trained with data very close to those used for the plot (see section 5). Discussions on the transferability of DM21 have started^99,100^ and will certainly continue. We found that, for example, scLH22t performs better than DM21 for the “multi-reference” MR16 subset of the W4–11 atomization energy benchmark.^72^ Returning to Figure 3, the rationally constructed scLHs and scRSLHs, but also the KP16/B13 functional also escape the zero-sum game to a significant extent. scLHs still exhibit somewhat larger delocalization errors than the scRSLHs, where ωLH23tdE-D4 stands out. KP16/B13-D4 has slightly lower FSEs than the latter scRSLH but somewhat larger delocalization errors.

**Figure 4:**
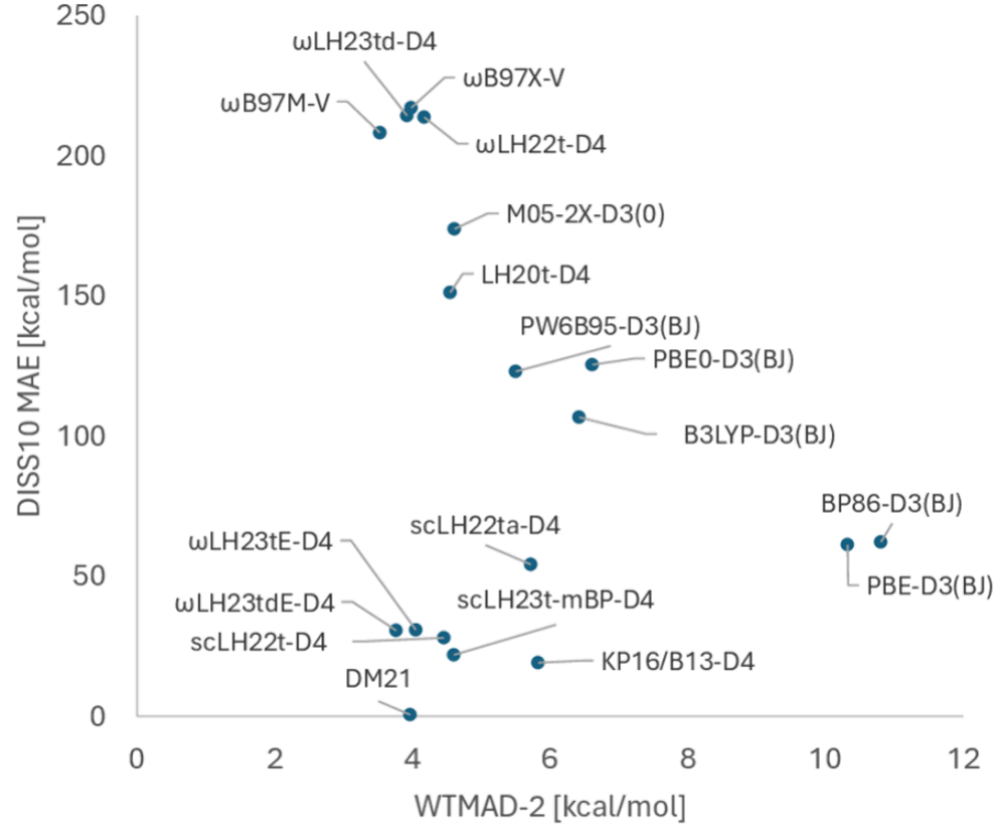
Evaluation of the zero-sum game for different functionals between DISS10 MAEs as FSE measure and the GMTKN55 WTMAD-2^60^ as a measure of performance for weakly correlated situations.

For a wider evaluation of FSEs vs performance for weakly correlated systems, Figure 4 plots the DISS10 MAEs^72^ against the overall weighted mean absolute deviations (WTMAD-2) of the large GMTKN55 test suite^60^ for the same set of functionals. GMTKN55 largely represents weakly correlated main-group systems. Most functionals again roughly fall on a line with negative slope indicating a zero-sum game, while scLHs, scRSLHs (including DM21) and KP16/B13-D4 clearly deviate from this line. While ωLH23td-D4 with DE- but without sc-corrections comes relatively close to the best-performing RSH ωB97M-V for GMTKN55, both of these functionals have very large FSEs. ωLH23tdE-D4 retains the excellent GMTKN55 performance while improving significantly on FSEs. It even improves somewhat over DM21 for GMTKN55 while still exhibiting more significant FSEs. KP16/B13-D4 has small FSEs but falls behind somewhat for GMTKN55. It clearly provides some escape from the zero-sum game as well but remains more difficult to use in routine applications than the scRSLHs or scLHs (see section 2.4 above).

**Figure 5:**
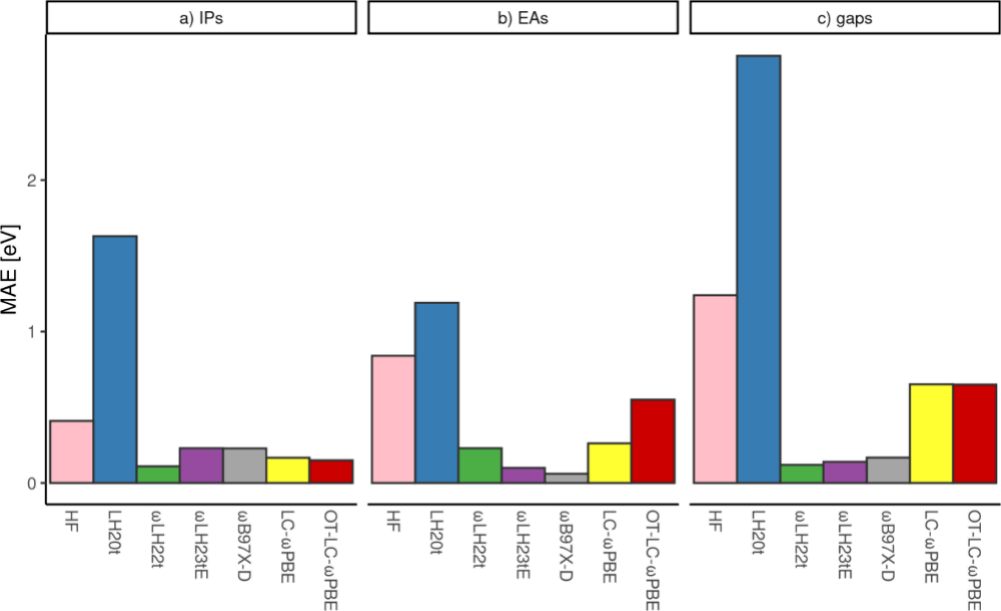
MAEs of IPs, EAs and fundamental gaps (in eV) of a series of oligoacenes (from benzene to hexacene) computed from HOMO and LUMO energies with different functionals^2,80,96^ compared to CCSD(T) reference data. Note that ωLH23tE without DE-corrections is equivalent to ωLH23tdE for the closed-shell systems studied.

We indicated above that ωLH22t provides outstanding HOMO and LUMO energies for a wide variety of chromophores to estimate IPs, EAs and fundamental gaps from Koopmans’ theorem (used in a generalized Kohn–Sham framework),^96^ competitive with optimally tuned RSHs but without their disadvantages. The fact that the scRSLHs derived from ωLH22t retain this excellent performance^2^ is a further important indication that these functionals escape the zero-sum game. As one of many examples from ref (2), Figure 5 shows the MAEs for the IPs, EAs, and fundamental gaps of a series of oligoacenes,^80^ obtained from the HOMO and LUMO energies, against CCSD(T) reference data. Clearly, ωLH23tE (which is equivalent to ωLH23tdE for closed-shell systems) is still competitive with ωLH22t and with the best tuned or untuned RSHs, in spite of its reduced FSEs due to the incorporation of sc-terms.

The scLHs and the ωLH22t RSLH have now been implemented for NMR nuclear shieldings^4^ and the related molecular magnetizabilities.^63^ For the latter case LHs dominate the overall best-performing functionals in a larger evaluation against CCSD(T) benchmark data. ωLH22t was also in this group, and scLHs actually provided the overall best performance.^63^ Most notably, they also exhibited excellent performance for the static-correlation case O_3_, which is usually excluded from the overall statistical evaluations. A detailed study of scLHs for two extended benchmark sets of main-group NMR shielding/shifts (NS372)^53^ and 3d transition-metal shifts (TM70)^62^ also indicated improvements for situations with appreciable static correlation, mostly for scLHs without damping in their *q*_*AC*_(**r**) functions.^4^ When used with the proper current-density response, the scLH22ta and scLH21ct-SVWN-m functionals were the overall best-performing LHs for both test sets and among the best-performing functionals overall. For TM70, the improvement due to sc-terms was most notable in the ^53^Cr subset. scLHs with damping terms provided less notable improvements.^4^

**Figure 6:**
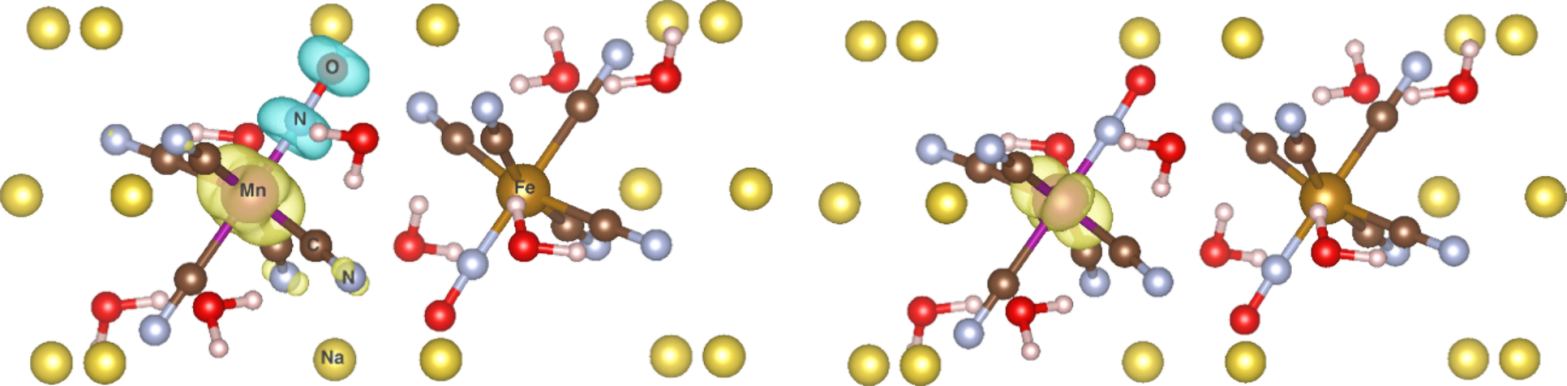
Introduction of “DE-corrections” into the td-LMF of ωLH23td reduces unphysical spin polarization (cf. negative spin density on NO ligand) and thus spin contamination for cluster-embedded [Mn(CN)_5_NO]^2–^ compared to ωLH22t. Figure adapted from ref (3), published 2024, CC-BY 4.0.

In a separate study, scLHs and scRSLHs were investigated for the question of spin-symmetry breaking and hyperfine couplings (HFCs) in a set of open-shell manganese complexes.^3^ Indeed, sc-terms reduce spin contamination and thereby improve dipolar HFCs compared to the underlying LHs or RSLHs, again most strongly without damping in *q*_*AC*_(**r**). Notably, however, the open-shell DE correction terms of scRSLHs like ωLH23tdE (see above), or even without sc-terms in ωLH23td, are even more effective in this context (see Figure 6 for the spin-density distribution in cluster-embedded [Mn(CN)_5_NO]^2–^). Interestingly, they operate by locally enhancing EXX admixture near the metal center and thereby reducing spin delocalization onto the ligands.^3^ In contrast, sc-terms reduce EXX admixture at crucial ligand atoms and thereby have a similar overall effect on spin-symmetry breaking.

## 7 Concluding Remarks and Future Directions

Approximate density functionals based directly on the local exact-exchange energy density offer unprecedented avenues toward an ever more accurate treatment of a wide variety of systems in chemistry, solid-state and material science, and adjacent fields. Further improvements can be expected from more refined strong-correlation terms and generally from more sophisticated local mixing functions, from the correlation functional and, in the case of scRSLHs, from the details of range separation (e.g., the possibility of using local range-separation functions^101^). Machine learning, such as used in the development of DM21,^97^ can play an important role in this endeavor, but rational constructions as described here help to root the resulting density functionals in an understandable physical picture. We also expect such rationally designed functionals to have advantages regarding efficient implementations and/or SCF convergence.^98^

Ultimately, the aim is to construct functionals that exhibit high accuracy in different regions of a molecule, solid, or complex composite system. This may pertain to different properties probing the core, valence or asymptotic regions. But it may also apply to different spatial parts in large multicomponent systems. A recent example is the application of a simple first-generation LH to improve the match between the frontier orbitals of a TiO_2_ surface and an organic dye in a cluster model for a dye-sensitized solar cell.^102^ One may extend, for example, such a vision to the application of the above-mentioned scRSLHs or of future functionals to charge transfer of an organic dye adsorbed on a transition-metal surface. It is clear that an accurate treatment of such a system requires simultaneously small delocalization errors within the dye and small FSEs within the metal (where long-range EXX admixture indeed often may be detrimental). The same holds if we want to model other types of chemical reactions or physical processes of complex systems, e.g., catalysis on transition-metal surfaces or clusters. Given that post-Hartree–Fock *ab initio* methods or DFT-like approaches based on multiconfigurational wave functions will be difficult to apply in such cases, the extension of KS DFT to such areas is clearly desirable.
